# Transcriptome Analysis Reveals Dynamic Gene Expression Profiles in Porcine Alveolar Macrophages in Response to the Chinese Highly Pathogenic Porcine Reproductive and Respiratory Syndrome Virus

**DOI:** 10.1155/2018/1538127

**Published:** 2018-04-29

**Authors:** Nanfang Zeng, Cong Wang, Siyu Liu, Qi Miao, Lei Zhou, Xinna Ge, Jun Han, Xin Guo, Hanchun Yang

**Affiliations:** Key Laboratory of Animal Epidemiology of the Ministry of Agriculture, College of Veterinary Medicine and State Key Laboratory of Agrobiotechnology, China Agricultural University, Beijing, China

## Abstract

Porcine reproductive and respiratory syndrome virus (PRRSV) is one of the most economically important swine pathogens and causes reproductive failure in sows and respiratory disease in growing pigs. PRRSV mainly infects porcine alveolar macrophages (PAMs), leading to the subversion of innate and adaptive immunity of pigs. The transcriptome analysis of gene expression profiles in PRRSV-infected PAMs is essential for understanding the pathogenesis of PRRSV. Here we performed next-generation RNA sequencing and a comprehensive bioinformatics analysis to characterize the dynamic transcriptome landscapes in PAMs following PRRSV infection. Totally 38222 annotated mRNAs, 12987 annotated long noncoding RNAs (lncRNAs), and 17624 novel lncRNAs in PRRSV-infected PAMs were identified through a transcripts computational identification pipeline. The differentially expressed mRNAs and lncRNAs during PRRSV infection were characterized. Several differentially expressed transcripts were validated using qRT-PCR. Analyses on dynamic overrepresented GO terms and KEGG pathways in PRRSV-infected PAMs at different time points were performed. Meanwhile the genes involved in IFN-related signaling pathways, proinflammatory cytokines and chemokines, phagocytosis, and antigen presentation and processing were significantly downregulated, indicating the aberrant function of PAMs during PRRSV infection. Moreover, the differentially and highly expressed lncRNA XR_297549.1 was predicted to both cis-regulate and trans-regulate its neighboring gene, prostaglandin-endoperoxide synthase 2 (PTGS2), indicating its role in inflammatory response. Our findings reveal the transcriptome profiles and differentially expressed mRNAs and lncRNAs in PRRSV-infected PAMs* in vitro*, providing valuable information for further exploration of PRRSV pathogenesis.

## 1. Introduction

Porcine reproductive and respiratory syndrome virus (PRRSV) has been one of the most economically significant swine pathogens worldwide for over two decades since it was first recognized in Europe in 1991 and independently in the USA in 1992 [1, 2]. The disease (PRRS) caused by this virus is characterized by reproductive failure in pregnant sows and respiratory disease in all stages of pigs [3, 4], leading to huge economic losses to global swine industry [5, 6]. The PRRSV is an enveloped, single-stranded, positive-sense RNA virus, belonging to the genus* Arterivirus* within the family Arteriviridae in the order Nidovirales [7]. Globally, PRRSV is divided into the European type (genotype 1) and North American type (genotype 2) based on genetic and antigenic differences [8–11]. It is newly proposed that the virus is classified into the genus* Porartevirus *of the family Arteriviridae and has two species (PRRSV1 and PRRSV2) (https://talk.ictvonline.org/ictv-reports/ictv_online_report/). The rapid evolution and variation of PRRSV result in the emergence and prevalence of novel strains in the field. Particularly, the Chinese highly pathogenic PRRSV (HP-PRRSV) characterized by a discontinuous 30-amino-acid deletion in nonstructural protein 2 (nsp2) brought about an unparalleled, large-scale, atypical PRRS outbreak in 2006, causing tremendous economic losses to the swine industry in China [12, 13]. Our previous studies have revealed that the nsp9 and nsp10 together contribute to the increased replication efficiency and fatal virulence for piglets of the Chinese HP-PRRSV [14]. However, the mechanisms underlying the dynamic cellular responses of porcine alveolar macrophages (PAMs) during HP-PRRSV infection have not been fully elucidated yet.

PRRSV has a very restricted tropism for cells of the monocytic lineage, and the fully differentiated PAMs are primary target cell for PRRSV infection [15]. It is of great significance to focus on PAMs in the study of PRRSV, because they play essential roles in lung tissue homeostasis, early pathogen recognition, initiation of the local immune responses, and resolution of inflammation [16]. To date, a considerable number of transcriptomic experiments have been conducted to reveal the gene expression profiles of PRRSV-infected PAMs either in an* in vitro* infection model or* in vivo* challenge model [17–24]. Although the* in vivo* model enabled us to evaluate the overall effects of PRRSV infection on the respiratory tract, the* in vitro* model helps us to assess the direct impacts of PAMs in response to PRRSV infection independently of the respiratory immune system. PAMs have been shown to present distinct gene expression profiles at different time points during PPRSV infection, indicating the dynamic interaction between PRRSV and PAMs at the transcriptional levels [17, 25]. Moreover, it has been illustrated that the transcriptome differences of PAMs existed in response to PRRSV strains with divergent virulence [18, 19], expanding the understanding of unique performance of defined PRRSV strain in PAMs at the transcriptional level [26]. However, majority of the studies mentioned above were performed using either microarrays or Digital Gene Expression (DGE) tag profiling with some limitations. The next-generation high-through RNA sequencing (RNA-Seq) is helpful for us to screen out large amounts of genetic information in model organisms [27]. RNA-Seq is able to provide enormous amounts of sequence data, usually tenfold or one hundredfold greater than those produced using traditional Sanger sequencing technology. In addition to protein-coding gene expression, RNA-Seq data can be used not only to identify transcription start site (TSS), splicing variants, and differential promoter usage, but also to reveal the expression profiles of annotated and novel long noncoding RNAs (lncRNAs), which are one of the newly emerging RNAs and of intriguing interest in recent years [27–29]. LncRNAs are arbitrarily defined as RNA molecules of greater than 200 nucleotides in length with poor protein-coding capacity. Compared to mRNAs, lncRNAs are more cell type-specific, less expressed, and less well-conserved [30, 31]. Broadly, lncRNAs can be classified into several categories, including precursor transcripts, enhancer-associated RNAs (eRNAs), and transcripts overlapping annotated genes in sense or antisense, as well as those that are self-contained transcription units within the genomic interval between two protein-coding genes. The precise sequence and structure of an lncRNA probably determine the number and type of protein or RNA that it interacts with. Through these interactions, several lncRNAs have been described to regulate gene expression in a variety of cellular processes, including innate and adaptive immunity [32, 33]. By using RNA-Seq, many viruses, such as enterovirus, influenza virus, human immunodeficiency virus (HIV), hepatitis B and C viruses, and the SARS coronavirus, have been shown to induce the expression alteration of lncRNAs [34–42]. The lncRNA signature is considered to be a mixture of transcripts by both virus infection and cellular countermeasures, reflecting another aspect of virus-host interaction besides differences in protein-coding gene expression.

In the present study, we identified a large number of expressed transcripts, especially mRNAs and lncRNAs, from PAMs infected with HP-PRRSV JXwn06 at different time points, using the Illumina HiSeq 2500 sequencing platform. In addition to the differentially expressed genes (DEGs) using both Gene Ontology (GO) database and Kyoto Encyclopedia of Genes and Genomes (KEGG) database, we also unraveled the potential role of essential lncRNAs involved in PRRSV infection in PAMs. Although a comprehensive understanding of differential posttranscriptional and posttranslational responses in PAMs remains to be determined, the data generated from our study contribute to a better understanding of the roles of PRRSV-PAMs interaction in the pathogenesis of PRRSV at the transcriptional level.

## 2. Methods and Material

### 2.1. Cells and Virus

PAMs were prepared from 6-week-old healthy landrace piglet as previously described [1, 43]. The piglets were purchased from Beijing Center for SPF Swine Breeding and Management that is free of PRRSV, porcine circovirus type 2 (PCV2), classical swine fever virus (CSFV), pseudorabies virus, swine influenza virus, and* Mycoplasma hyopneumoniae* infection. The lung lavage fluid was collected from the lungs of euthanized piglets and washed ten times with PBS supplemented with 2% fetal bovine serum (Fisher Scientific, Waltham, MA, USA). The cell pellets were resuspended and mixed with prechilled GIBCO RPMI-1640 medium (Fisher Scientific) containing 40% fetal bovine serum (FBS) (Hyclone Laboratories Inc., South Logan, UT, USA). The number of the prepared PAMs reached 10^8^–10^9^/ml with >95% viability. Aliquots of PAMs were frozen and stored in liquid nitrogen before use. The viability of PAMs was determined to be 85%–90% by trypan blue dye exclusion. PAMs were maintained in GIBCO RPMI-1640 medium, with 10% FBS, 100 mg/ml kanamycin, 50 U/ml penicillin, 50 mg/ml streptomycin, 25 mg/ml polymixin B, and 1 mg/ml fungizone at 37°C, 5% CO_2_.

The 8th passage virus of a Chinese highly pathogenic PRRSV JXwn06 (GenBank accession number EF641008) was used in this study [44]. PAMs were cultured for 48 h at 37°C, 5% CO_2_ in cell culture dish (Corning Inc., Corning, NY, USA) at a density of 5 × 10^7^ cells/dish with RPMI-1640 medium, and the nonadherent cells were moved by gentle washing with RPMI-1640 medium prior to inoculation. The cells were inoculated with PRRSV JXwn06 at a multiplicity of infection (MOI) of 10. After adsorption for 1 h, the inoculum was removed, and the cells were washed with PBS and then supplemented with RPMI-1640 medium containing 5% FBS. At 6 h, 9 h, or 12 h postinoculation (hpi) in each PRRSV-infected group (P_V6_, P_V9_, and P_V12_ group), the supernatant was discarded, and the cells were collected and TRIzol (Fisher Scientific) was added immediately. Then cells were cryopreserved in liquid nitrogen for further transcriptomic analysis. Similarly, the uninfected PAMs served as mock-infected cells. All the infection experiments were performed in duplicate.

### 2.2. Whole Transcriptome Library Preparation and Sequencing

Total RNAs were extracted from PRRSV-infected PAMs and mock-infected PAMs using TRIzol according to the manufacturer's instructions. RNA library construction and sequencing were performed by RiboBio Co. Ltd. (Guangzhou, China). Briefly, prior to library construction, RNA purity was checked using the ND-1000 Nanodrop (Fisher Scientific). Each RNA sample had an A260 : A280 ratio above 1.8 and A260 : A230 ratio above 2.0. RNA integrity was evaluated using the Agilent 2200 TapeStation (Agilent Technologies, Santa Clara, CA, USA). The samples from PRRSV-infected PAMs at 24 hpi were abandoned because of their RIN value below 7.0. The rRNAs were then removed from total RNA using Epicentre Ribo-Zero rRNA Removal Kit (Illumina, San Diego, CA, USA) and fragmented to approximately 200 bp. Subsequently, the purified RNAs were subjected to first-strand and second-strand cDNA synthesis followed by adaptor ligation and enrichment with a low-cycle according to instruction of TruSeq® RNA LT/HT Sample Prep Kit (Illumina). The purified library products were evaluated using the Agilent 2200 TapeStation and Qubit®2.0 (Fisher Scientific) and then diluted to 10 pM for cluster generation in situ on the HiSeq 2500 pair-end flow cell followed by sequencing (2 × 100 bp) on HiSeq 2500 (Illumina).

### 2.3. RNA-Seq Data Analysis

The raw sequencing data (raw reads) were preserved in FASTQ format. Clean reads were obtained by removing adaptors, reads of the unknown base with more than 10%, and those with low quality from the raw reads. The corresponding software Cutadapt 1.8.1 and software NGSQC Toolkit (v2.3.3) were employed [45, 46]. Clean data of high quality were then aligned to the* Sus scrofa* genome assembly (*Sus scrofa *10.2) using TopHat2 (v2.0.9) [47]. The transcriptome of each sample was assembled from the mapped reads by Cufflinks (v2.1.1) [48]. Transcripts, including annotated mRNAs and lncRNAs, were identified according to the* Sus scrofa* genome assembly (*Sus scrofa *10.2). For novel lncRNA identification, the first step was conducted to filter the known lncRNAs and other known non-lncRNA annotations, including protein-coding genes, microRNAs, tRNAs, miscRNA, rRNAs, and pseudogenes. Then transcripts that are with less than 200 nt or single-exonic, which might result from potential DNA contamination, were filtered. Those filtered transcripts with predicted open reading frame (ORF) of <300 nt were selected for further coding potential calculation. By using Coding Potential Calculator (CPC) (version 2) [49, 50], all transcripts with coding potential score of <−1 were discarded. To exclude protein-coding transcripts thoroughly, the selected transcripts in all three possible reading frames were translated and mapped to the known protein domains cataloged in the Pfam database [51] using Pfam Scan (v1.3). Finally, the remaining transcripts were considered reliably as expressed novel lncRNAs.

### 2.4. Differential Expression Analysis

For each sample, the read counts of each transcript were normalized to the length of individual transcript and to the total mapped fragment counts and expressed as reads per kilobase per million mapped (RPKM) reads of both mRNAs and lncRNAs. By using DESeq, the mRNA and lncRNA differential expression analyses were performed for all pairwise comparisons including P_V6_ versus P_M_, P_V9_ versus P_M_, P_V12_ versus P_M_, P_V9_ versus P_V6_, and P_V12_ versus P_V9_. Moreover, the genes in all PRRSV-infected groups (P_V_) were compared with the genes in mock-infected group (P_V_ versus P_M_), and the differentially expressed genes with the same change tendency during PRRSV infection were identified. A corrected *p* value < 0.05 by Student's *t*-test with Benjamini-Hochberg FDR adjustment was used as the cut-off for significant differentially expressed genes.

### 2.5. GO and KEGG Enrichment Analysis

Both GOseq R package (1.18.0) [52] and KOBAS software (2.0) [53] were used for GO and KEGG pathway analyses. Differentially expressed protein-coding genes from all pairwise comparisons were used for enrichment analysis to detect overrepresented functional terms present in the genomic background.

### 2.6. Prediction of the Function of lncRNAs

Most of the annotated lncRNAs in current databases have not been functionally annotated yet. Prediction of their functions was performed based on their related cis- and trans-target mRNAs which have been functionally well annotated. Potentially cis-regulated target genes were defined as protein-coding genes within 10 kb in genomic distance from the lncRNA and potentially trans-regulated target genes using RNAplex (G < −20) (version 0.2) [54].

### 2.7. Quantitative Real-Time PCR

Total RNAs were extracted using TRIzol (Fisher Scientific) following the manufacturer's instructions. The cDNA was reverse-transcribed from 1 *μ*g of total RNA using a Quant One-Step RT-PCR Kit (TIANGEN, Beijing, China). All qRT-PCR primers synthesized by GenePharma (Shanghai, China) (Table 1) were verified to produce specific PCR product and react efficiently. All qRT-PCR reactions were performed on a 7500 real-time PCR system (Fisher Scientific) using Quant One-Step qRT-PCR Kit (TIANGEN) with technical triplicates. Relative quantification of target genes was performed using the −2^−ΔΔCt^ method with PPIA as a reference gene.

### 2.8. Statistical Analysis

The data from qRT-PCR were shown as means ± standard deviations (SD). The Graphpad Prism software (version 5.0) was used to determine the significance of the variability among different groups by two-way ANOVA test of variance. A *p* value < 0.05 was considered to be statistically significant.

## 3. Results

### 3.1. Transcriptome Analysis of PAMs in Response to PRRSV

**Figure 1:**
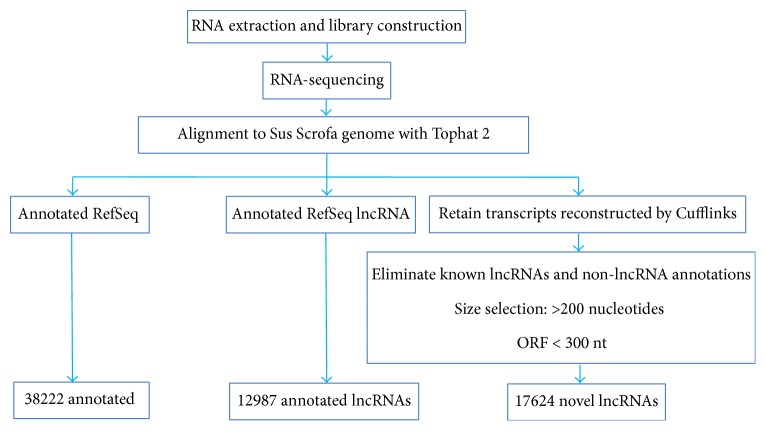
The bioinformatics pipeline for the systematic identification of mRNAs and lncRNAs in PAMs.

PAMs were infected with PRRSV JXwn06 at a multiplicity of infection (MOI) of 10 to ensure that majority of cells become infected and the infection is more synchronized among PAMs [55]. Besides two mock-infected samples, every two PRRSV-infected samples were collected at 6, 9, and 12 hpi, respectively, and pooled for library construction and sequencing. Total RNAs were prepared from PRRSV-infected PAMs and mock-infected PAMs. Each sample was then subjected to Illumina-based RNA sequencing. After cleaning and quality testing, clean reads were screened out from raw sequencing data (raw reads) and mapped to the* Sus scrofa* genome assembly (*Sus scrofa* 10.2) [56]. With a transcripts computational identification pipeline, totally 38222 annotated mRNAs, 12987 annotated long noncoding RNAs (lncRNAs), and 17624 novel lncRNAs in PRRSV-infected PAMs were identified (Figure 1). The corresponding data statistics of each sample was listed in Table S1.

### 3.2. Distinct Expression Profiles of PAMs in Response to PRRSV at Different Time Points

**Figure 2:**
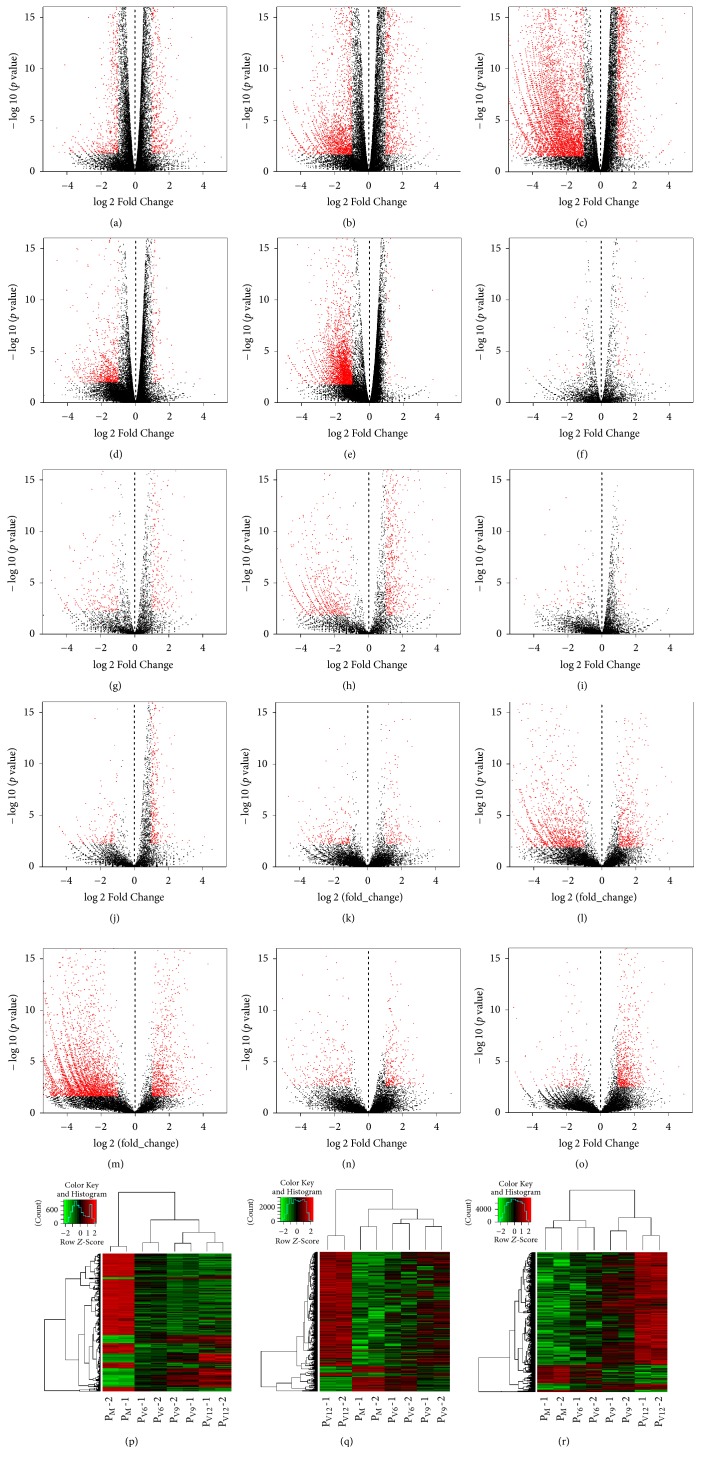
*Gene expression profiles distinguishing different groups*. Shown is the volcano plot of differentially expressed genes between each comparison group, including the mRNAs between P_V6_ and P_M_ (a), P_V9_ and P_M_ (b), P_V12_ and P_M_ (c), P_V9_ and P_V6_ (d), and P_V12_ and P_V9_ (e), the annotated lncRNAs between P_V6_ and P_M_ (f), P_V9_ and P_M_ (g), P_V12_ and P_M_ (h), P_V9_ and P_V6_ (i), and P_V12_ and P_V9_ (j), and the novel lncRNAs between P_V6_ and P_M_ (k), P_V9_ and P_M_ (l), P_V12_ and P_M_ (m), P_V9_ and P_V6_ (n), and P_V12_ and P_V9_ (o). Red dots denote the expressed genes with a greater than 2-fold expression change and FDR-corrected *p* value < 0.05. Black dots denote the genes that were expressed comparably in comparison groups. (p, q, r) Unsupervised hierarchical clustering of the expression profiles of differentially expressed mRNAs, annotated lncRNAs, and novel lncRNAs with the same change trend in PRRSV-infected groups in comparison to mock-infected groups, respectively.

With the threshold of |log⁡2(fold  change)| ≥ 1 and a false discovery rate- (FDR-) corrected *p* value < 0.05, the numbers of differentially expressed mRNAs and lncRNAs were identified when mock-infected PAMs (P_M_) group was compared with PRRSV-infected PAMs (P_V6_, P_V9_, and P_V12_ group, resp.) (Table 2). Meanwhile, distinct expression numbers of annotated and novel lncRNAs were also characterized (Table 2). To analyze the dynamic alteration of transcripts in PRRSV-infected PAMs at different time points, the volcano plots were conducted to describe each differentially expressed transcript in each comparison group, including P_V6_ versus P_M_, P_V9_ versus P_M_, P_V12_ versus P_M_, P_V9_ versus P_V6_, and P_V12_ versus P_V9_ (Figures 2(a), 2(b), 2(c), 2(d), 2(e), 2(f), 2(g), 2(h), 2(i), 2(j), 2(k), 2(l), 2(m), 2(n), and 2(o)). At 9 hpi, the number of upregulated mRNAs and lncRNAs was more than those of downregulated ones, indicating the activation status of PAMs in response to PRRSV. In addition, the transcripts with the same change tendency and significant differences during PRRSV infection were also screened out for further analysis (Figures 2(p), 2(q), and 2(r)). Based on the above results, further analyzing the potential role of these mRNAs and lncRNAs in the biological function of PAMs is required in order to expand the effect of PRRSV infection on PAMs* in vitro*.

### 3.3. Experimental Validation of Selected mRNAs and lncRNAs

**Figure 3:**
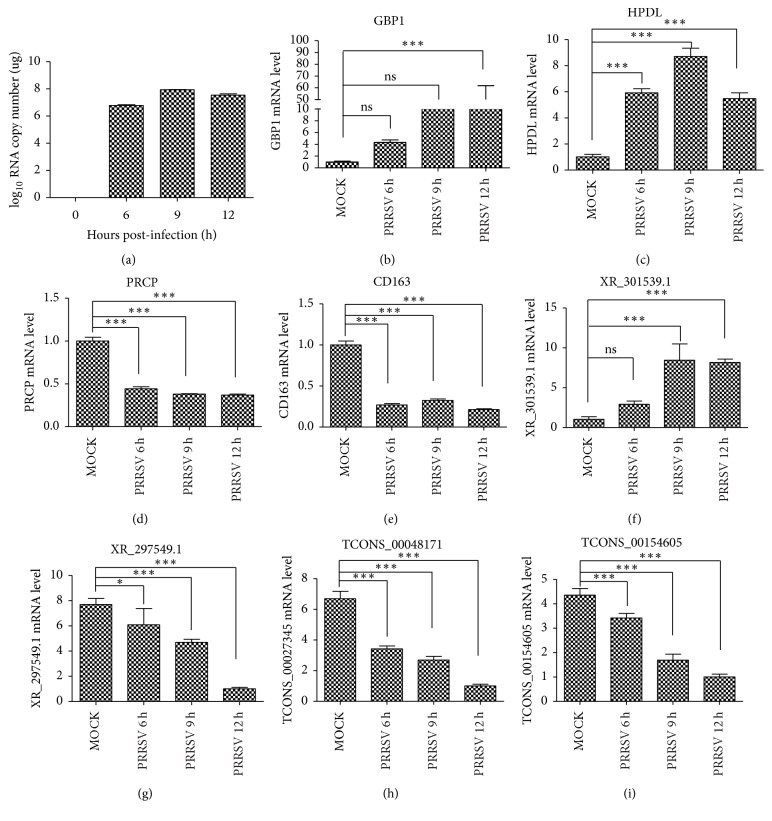
*qRT-PCR validation of PRRSV infection and differentially expressed genes in PAMs in response to PRRSV at different time points*. Shown are the mRNA level of PRRSV N gene (a), the expression patterns of multiple annotated mRNAs (b–e), the expression patterns of two annotated lncRNAs (f, g), and the expression patterns of two novel lncRNAs (h, i). qRT-PCR was performed by using the primers specific for corresponding gene. PPIA served as the reference gene. Error bars represent the standard error of three biological replicates. Asterisks indicate significant differences by Student's test (^*∗*^*p* < 0.05; ^*∗∗∗*^*p* < 0.001; ns, not significant).

The quantitative real-time PCR (qRT-PCR) analysis was performed with the respective primers and probes to analyze the relevant transcripts from our original samples used in deep-sequencing. Firstly, the mRNA levels of PRRSV N gene were examined in each sample to confirm the infection status of PAMs (Figure 3(a)). Then four annotated mRNAs including guanylate-binding protein 1 (GBP1), 4-hydroxyphenylpyruvate dioxygenase like (HPDL), prolylcarboxypeptidase (PRCP), and cluster of differentiation CD163, two annotated lncRNAs (XR-301539 and XR-297549.1), and two novel lncRNAs (TCONS-00048171 and TCONS-00154605) from each PRRSV-infected group and mock-infected group were selected for qRT-PCR analysis. The results showed that all of the transcripts exhibited similar change tendency following PRRSV infection (Figures 3(b), 3(c), 3(d), 3(e), 3(f), 3(g), 3(h), and 3(i)), consolidating the results obtained through RNA-Seq.

### 3.4. Characteristic Analysis of the Dynamic Gene Expression in PRRSV-Infected PAMs at Different Time Points

**Figure 4:**
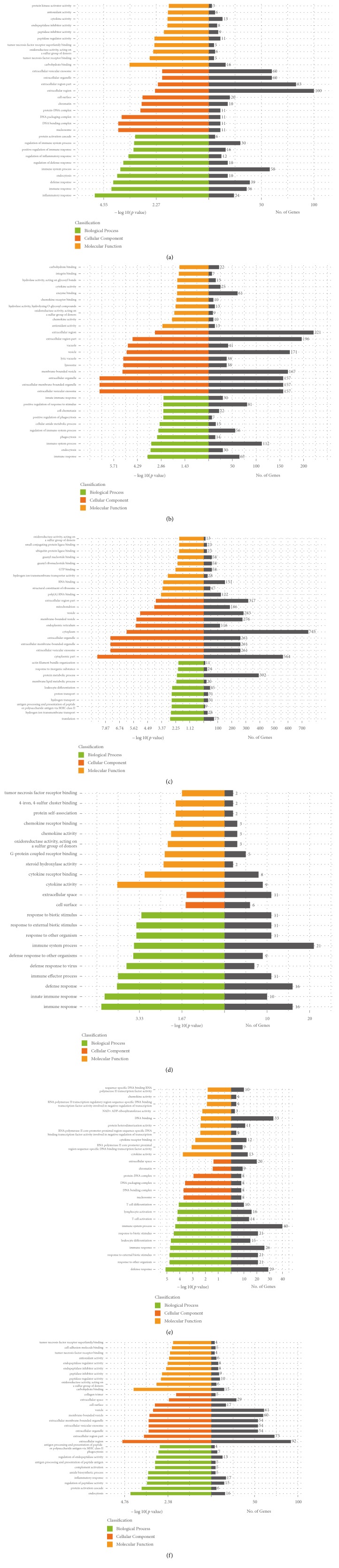
*Significant Gene Ontology (GO) annotations of differentially expressed genes*. The three main GO categories including biological process, cellular component, and molecular function were analyzed. Shown are the statistically overrepresented GO terms between P_V6_ and P_M_ group (a), P_V9_ and P_M_ group (b), P_V12_ and P_M_ group (c), P_V9_ and P_V6_ group (d), and P_V12_ and P_V9_ group (e). (f) The differentially expressed mRNAs with the same change trend in PRRSV-infected groups in comparison to mock-infected groups.

To obtain a comprehensive understanding of the dynamic gene expression profiles during PRRSV replication, protein-coding genes were further screened out with the threshold as follows: (i) |log⁡2(fold  change)| ≥ 1; (ii) FDR-corrected* p value* < 0.05; (iii) RPKM ≥ 1. Totally, the differentially expressed protein-coding genes, including 991 genes in P_V6_ group, 2892 genes in P_V9_ group, and 6208 genes in P_V12_ group, were characterized when compared with the P_M_ group. Eight hundred and five and 3067 genes were identified when compared between P_V9_ and P_V6_ groups or P_V12_ and P_V9_ groups, respectively. Moreover, 770 genes with the same change tendency during PRRSV infection were also classified and all of these genes were used for GO term and KEGG pathway enrichment analyses using specific* Sus scrofa* gene database as the background. GO analysis of the differentially expressed protein-coding genes between each PRRSV-infected group and mock-infected group were shown in Figures 4(a), 4(b), and 4(c). These differentially expressed protein-coding genes included biological process terms (inflammatory response, immune response, defense response, and endocytosis), cellular component terms (nucleosome, DNA bending complex, DNA packaging complex, and protein-DNA complex), and molecular function terms (carbohydrate binding, tumor necrosis factor receptor binding, oxidoreductase activity acting on a sulfur group of donors, and tumor necrosis factor receptor superfamily binding). Considering the dynamic gene expression profiles at different time points postinfection, further analysis of the genes with same change tendency at each time point postinfection was carried out (Figure 4(d)). Moreover, comparative analysis between P_V9_ and P_V6_ groups or between P_V12_ and P_V9_ groups was performed (Figures 4(e) and 4(f)).

**Figure 5:**
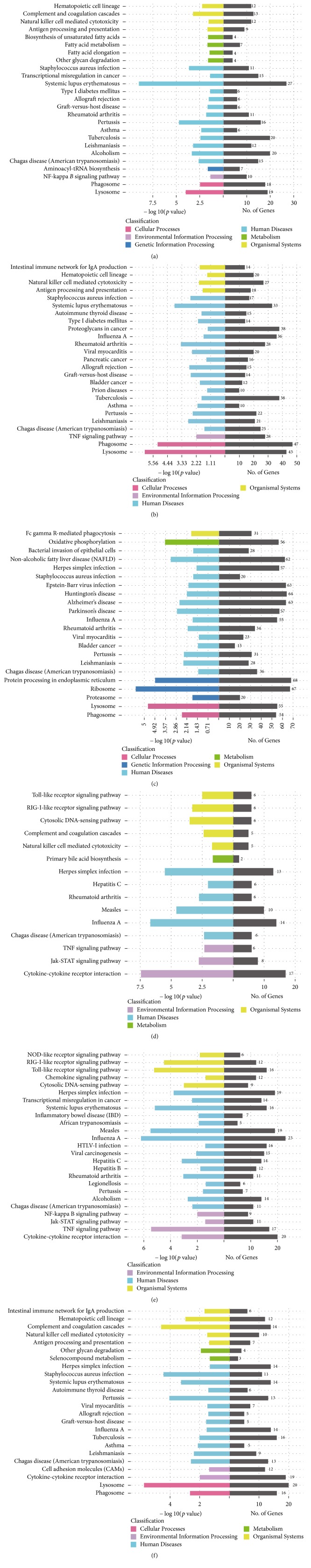
*KEGG pathway analysis of differentially expressed protein-coding genes*. Shown are the top overrepresented KEGG pathways between P_V6_ and P_M_ group (a), P_V9_ and P_M_ group (b), P_V12_ and P_M_ group (c), P_V9_ and P_V6_ group (d), and P_V12_ and P_V9_ group (e). (f) The differentially expressed mRNAs with the same change trend in PRRSV-infected groups in comparison to mock-infected groups. The pathways showing –log⁡10 (*p* value) > 1.3 are considered statistically significantly overrepresented.

KEGG pathway analyses of the differentially expressed mRNAs were performed between PRRSV-infected group and mock-infected group. The results revealed that these differentially expressed mRNAs were related to the phagosome, lysosome, and others during PRRSV infection. The specific overrepresented KEGG pathways at each time point were shown in Figures 5(a), 5(b), 5(c), and 5(d). In addition, P_V6_, P_V9_, and P_V12_ groups were compared with each other and overrepresented pathways were presented in Figures 5(e) and 5(f). Of note, during PRRSV infection, the overrepresented lysosome and phagosome pathways are of great interest for further analysis due to their essential roles in antiviral and antibacterial responses.

### 3.5. Characteristic Analysis of Key Genes Involved in PAMs Function during PRRSV Infection

PRRSV is shown to enter the host cell through receptor-mediated endocytosis [57]. Upon internalization, the viral genome is released into the cytoplasm to initiate transcription and replication. Recognition of viral nucleic acid by either cytosolic RIG-I-like receptors (RLRs) or endosomal Toll-like receptors (TLRs) leads to the initiation of antiviral signaling cascades, triggering the production of cytokines and chemokines [58–62]. The genes differentially expressed during PRRSV infection in Toll-like receptor signaling pathway and RIG-I-like receptor signaling pathway were listed in Table S2. Type I interferons are considered as key antiviral cytokines which trigger the activation of Janus kinase-signal transducer and activator of transcription (JAK-STAT) signaling pathway and expression of interferon-stimulated genes (ISGs) and related antiviral effectors [63]. For the transcription and replication, PRRSV has evolved multiple strategies to interfere with IFN-mediated signaling pathways and to block the action of ISGs with antiviral activity [64]. Our analyses on the genes altered in the downstream JAK-STAT signaling pathway showed that only PIK3R5 and PIK3CB were significantly suppressed in Pv9 group and Pv12 group (Table S2). By comparative analysis between PRRSV-infected and mock-infected groups, majority of the ISGs were significantly increased at 9 and 12 hpi (Table S3), implying the start of host antiviral immune response at 9 h following PRRSV infection.

Besides JAK-STAT signaling pathway, MAPK and NF-*κ*B are also essential signaling pathways activated during PRRSV infection [64]. Our analyses indicated that the transcriptome abundance of genes in NF-*κ*B signaling pathway had no significant alteration during PRRSV infection, while the genes involved in MAPK signaling pathway were significantly decreased (Table S2).

The transcription levels of the downstream cytokines and chemokines in PAMs during PRRSV infection were characterized. Interestingly, none of the proinflammatory cytokines genes, including interleukin-1*β* (IL-1*β*), IL-6, IL-8, IL12, and IL-18, were highly expressed in both PRRSV-infected and mock-infected PAMs. Although the mRNA level of IFN-*α* was very low upon PRRSV infection, the mRNA levels of IFN-*β* and IFN-*αω* were significantly upregulated at 9 hpi and 12 hpi compared with those in mock-infected groups.

The heterogeneous and plastic properties diversify the dynamic function of macrophages in response to different environmental stimuli. To date, multiple receptors, cytokines, chemokines, and metabolic factors have been used as potential biomarkers of different activation status of macrophages [65–67]. Although the transcription levels of these characteristic genes alone may not fully elucidate their protein expression or secretion status, the change tendency in response to PRRSV indeed reflects the polarized direction of macrophages to some degrees. Our results showed that the basal levels of most characterized receptors in M1, M2a, and M2c phenotypes were higher, and two M1-specific cytokines (IL-1*β* and TNF-*α*), one M1-specific metabolic factor PTGS2, one M2a-specific chemokine C-C motif chemokine ligand 23 (CCL23), and one M2a-specific and one M2b-specific chemokine C-X-C motif ligand (CXCL2) were expressed at high transcriptional level (Table S4). The gene expression quantities and change tendency of these biomarkers in PRRSV-infected PAMs indicated specific phenotypes at each time course upon PRRSV infection compared with the well-characterized classically activated macrophage (M1) and alternatively activated macrophages (M2) statuses. Of note, the proinflammatory cytokines and chemokines, including TNF-*α*, transforming growth factor-*β* (TGF-*β*), IL-1*β*, CCL3, CCL4, CCL23, CXCL14, and IL-6, were downregulated, indicating the anti-inflammatory property of PAMs after PRRSV JXwn06 infection* in vitro*. In addition, the suppression of TLR1, TLR4, and TLR8 in PRRSV-infected PAMs might weaken the recognition of PAMs for invading pathogens. An essential role of macrophages is to present the antigens to the corresponding immune cells through either the major histocompatibility complex I (MHC I) or MHC II pathway. Our previous studies have demonstrated that PRRSV has evolved to evade cytotoxic T lymphocyte (CTL) responses through nsp1*α*-mediated swine leucocyte antigen (SLA-1) proteasomal degradation [68]. KEGG enrichment analysis showed the overrepresented antigen processing and presentation pathways (Figure 5). Further characterization of expression level of these overrepresented genes revealed that multiple isoforms of SLA-I and SLA-II and key genes involved in antigen processing were remarkably suppressed during PRRSV infection (Table S5), further indicating the aberrant antigen processing and presentation ability of PAMs in response to PRRSV infection.

Phagocytosis and the subsequent clearance of exogenous pathogens in phagolysosome are another major function of macrophages. PRRSV infection has been shown to impair the phagocytic and microbicidal capacity of PAMs, increasing the susceptibility to bacterial infection [69, 70]. Our analyses discovered that a variety of genes involved in phagosome and Fc*γ*R-mediated phagocytosis pathways were significantly downregulated (Table S6). As lysosomes also participate in pathogen clearance process, we also analyzed the expression level of the related genes. Of note, a great variety of lysosomal acid hydrolases, including proteases, glycosidases, sulfatases, lipases, nucleases, and aspartylglucosaminidases, were significantly downregulated at the transcription level. Moreover, the lysosomal membrane proteins, in particular lysosomal-associated membrane protein 1 (LAMP1), also had the decreased transcription levels, suggesting that the lysosomal function of PAMs is impaired during PRRSV infection.

### 3.6. Function Prediction of Annotated lncRNAs of Interest

lncRNAs have been considered as important regulators of gene expression. Among them, several lncRNAs have been identified to regulate the proximal protein-coding genes in* cis*, while some of them have been characterized to control remote gene expression in* trans*. Although more and more lncRNAs are functionally annotated in* Homo sapiens* and* Mus musculus*, none of them have been functionally identified in* Sus scrofa* assembly. Our RNA-Seq analysis revealed that PRRSV infection could trigger the dynamic expression profiles of lncRNAs in PAMs. Here two upregulated annotated lncRNAs (XR_301539.1 and XR_301635.1) and three downregulated lncRNAs (XR_297549.1, XR_304346.1, and XR_299147.1) were selected for further functional analysis. Of them, only lncRNA XR_301539.1 and lncRNA XR_297549.1 are proximal to annotated genes, implying that they share the probability of cis-regulation activity. Furthermore, by using RNAplex software, PTGS2 and TMEM254 were found to be the genes trans-regulated potentially by lncRNA XR_297549.1 and lncRNA XR_299147.1, respectively (Table 3). Of note, PTGS2 gene was also predicted to be both cis-regulated and trans-regulated by lncRNA XR_297549.1, showing a higher possibility of functional correlation of these transcripts. Together with the similar change tendency of lncRNA XR_297549.1 and PTGS2 gene (Table S4 and Table 3), it is proposed that the lncRNA XR_297549.1 possibly participates in the regulation of PTGS2 gene. Intriguingly, two lncRNAs have been annotated to approximate to PTGS2 gene in either* Homo sapiens *or* Mus musculus* [71]. Three splice variants of lincRNA-COX2 are proximal to the PTGS2 gene in* Mus musculus* [71], and p50-associated COX-2 extragenic RNA (PACER) is recognized as a contiguous antisense lncRNA in the upstream of PTGS2 mRNA start site in* Homo sapiens* [72]. Although the lncRNA XR_297549.1 in* Sus scrofa* is also in the upstream of PTGS2 gene, it shares dramatic difference in nucleotide sequence when compared with PACER and lincRNA-COX2. As cyclooxygenase-2 (COX-2), encoded by PTGS2 gene, is one of the key regulatory enzymes involved in the production of prostaglandins and other prostanoids and plays a key role in the regulation of viral replication and inflammatory response, it is of great interest to further validate the potential regulatory role of lncRNA XR_297549.1 on PTGS2 gene and PRRSV infection.

## 4. Discussion

PRRSV infection can trigger a cascade of cellular events and responses of PAMs, leading to a unique transcriptome landscape reflecting the characteristics of PRRSV-pig interaction. Recent advances in this field have revealed distinct gene expression profiles of PAMs in response to different strains of PRRSV. By using Affymetrix microarrays, the PRRSV Lelystad virus- (LV-) infected PAMs showed limited transcripts of differentially expressed and significantly upregulated IFN-*β* transcription at 9 hpi during the first round of virus replication, implying the initiation of cellular innate immune response [25]. Subsequently, the transcriptome changes of PAMs* in vitro* at 12 hpi were further compared between LV and European highly virulent strain Lena infections [18], and through RNA-Seq technology the previous information was consolidated and enriched. Considering the biological similarities but distinct serological properties between genotype 1 and genotype 2 PRRSV, serial analyses of gene expression (SAGE) libraries were conducted to investigate the reactome dynamics of PAMs in response to genotype 2 PRRSV with low pathogenicity* in vitro* [17], indicating the differentially expressed genes (DEGs) of PRRSV-infected PAMs at different time points postinfection. Moreover, transcriptomic comparison of PAMs from resistant and susceptible pigs after PRRSV challenge showed that the genes enriched in activation of leukocyte extravasation and in suppression of apoptosis contributed to the resistance to PRRSV infection [21, 22]. The microRNA transcriptome of PAMs in response to different strains of PRRSV has expanded our understanding of cellular noncoding RNAs in PRRSV infection [73, 74]. A latest study analyzed the lncRNA expression profiles of PAMs in response to different pathogenic PRRSV strains, further expanding our understanding of predicted lncRNAs and their potential role in antiviral immune response [19].

In the present study, we focused on the dynamic transcriptome landscape of PAMs at different time points during PRRSV infection. The RNAs isolated from PRRSV-infected PAMs at 24 hpi were not used for further study as they could not meet the criteria of RNA quality control. As well known, the binding of the candidate receptors on the surface of PAMs initiates PRRSV infection through endocytosis [64, 75–78]. The pattern recognition receptors, including endosomal TLRs and RLRs, recognize viral nucleic acid and trigger the activation of innate antiviral responses in the course of infection [58–62]. Interaction between IFN-mediated innate immune response and PRRSV has been extensively studied in recent years [54]. Multiple proteins of PRRSV have been well-characterized to antagonize innate immune responses through distinct mechanisms [79–83]. Therefore, the genes involved in innate immune response, including the pattern recognition receptors and downstream cascades, were analyzed, and multiple essential genes were identified for further validation in our study. In accordance with previous studies [18], we also discovered that IFN-*β*, not IFN-*α*, was significantly increased at the transcriptional level at 9 hpi. Together with the upregulation of known interferon-stimulated genes (ISGs) at 9 hpi, it is proposed that innate immune responses of PAMs against PRRSV infection started at that time. As JAK-STAT signaling pathway is well known to mediate extracellular IFN signals to nucleus, resulting in ISGs expression and production of antiviral effectors, we compared the genes involved in JAK-STAT signaling pathway between PRRSV-infected and mock-infected PAMs. Intriguingly, most of the genes were remarkably decreased at all time points postinfection, indicating the decreased sensitivity of JAK-STAT signaling pathway in response to upstream cytokine-cytokine receptor interactions. A variety of antiviral ISGs have been identified and characterized to inhibit virus infection at diverse stages of virus life cycle. On the contrary, viruses have evolved to counteract this effect through different mechanisms. The nsp2 of PRRSV has been shown to antagonize the antiviral effect of ISG15 and ISGylation [84]. In addition, the interaction of PRRSV nsp3 with IFITM1, a broad-spectrum antiviral protein, has been confirmed to contribute to its degradation through proteasome-dependent manner [85]. Our analyses showed that most of the well-defined antiviral ISGs were upregulated at least twofold at 9 hpi. Therefore, whether PRRSV has evolved to counter antiviral proteins through one-to-one correlation manner or lower the global amounts of antiviral effectors generally needs further investigation.

Macrophages with multiple functions and heterogeneity play essential roles in both innate and adaptive immunity of host. M1 macrophages are induced by TLR ligands and IFN-*γ*, while M2 macrophages can be further categorized into 3 subtypes: IL-4/13-activated M2a, immune complex-activated M2b, and IL-10-deactivated M2c [76, 86, 87]. The specialized activation status of macrophages, characterized by their expression of cell surface markers, secreted cytokines and chemokines, and transcription and epigenetic pathways, exerts diverse functions in the regulation of inflammation, tissue repair, T- and B-cell proliferation, phagocytosis, and antimicrobial activity against distinct pathogens [65–67]. In our study, PAMs were cultivated for 48 h to make them more susceptible to PRRSV. We tried to find some clues about polarization from the change tendency of phenotype biomarkers; however, most of the biomarkers in all kinds of phenotypes (M1, M2a, M2b, and M2c) were downregulated at the transcriptional level, indicating the polarized direction of PAMs in response to PRRSV JXwn06 is not related to classical M1 or M2 phenotype. Similar to the previous study [14], some anti-inflammatory cytokines, like IL-10, were upregulated at 12 hpi. Most of the proinflammatory cytokines and chemokines were downregulated. Similar to the previous studies [18, 23], the expression levels of genes involved in antigen processing and presentation pathways were significantly downregulated. Multiple isoforms of SLA-I were significantly downregulated, implying that PRRSV has evolved to downregulate SLA-I expression not only through nsp1*α*-mediated proteasomal degradation of already expressed SLA-1 proteins [66], but also through blocking their new biosynthesis at the transcriptional level. Intriguingly, our study showed that two key genes involved in antigen processing and presentation [88, 89], the endoplasmic reticulum luminal glycoprotein 57 (ERp57) and interferon-*γ*-inducible lysosomal thiol reductase (GILT), were remarkably suppressed during PRRSV infection. Whether the downregulation of ERp57 and GILT at transcriptional level is alternative evasion mechanism of PRRSV requires further investigation.

PRRSV infection is shown to impair the phagosomal maturation of PAMs [90]. Similar to the previous study [18], our analyses also identified that a great variety of phagocytosis-promoting receptor genes were significantly decreased, implying that PRRSV infection can impair the receptor-mediated uptake process of phagocytosis of PAMs. Moreover, the genes involved in major lysosomal membrane components and lysosomal acid hydrolases were significantly downregulated, indicating the impaired function of lysosomes and phagolysosomes in PRRSV-infected PAMs. Several components of V-type adenylpyrophosphatase (ATPase) determining the acidic condition in phagolysosomes were also negatively regulated, further confirming the aberrant phagocytic function of PAMs following PRRSV infection.

Badaoui et al. have also investigated the dynamic interaction between PRRSV and PAMs at the transcriptome level and screened out the differentially expressed genes [18]. Different from their study in filtering condition of differentially expressed genes, our study indicated that the tumor necrosis factor (TNF) signaling pathway was one of the most significantly overrepresented pathways, especially in comparison between P_V9_ and P_M_ groups. Liang et al. also showed that the TNF signaling pathway was enriched in a highly pathogenic PRRSV-infected PAMs derived from large white piglets* in vivo* [22]. Combined with our previous study that HP-PPRSV and low pathogenic PRRSV (LP-PRRSV) infection exhibited a differential TNF-*α* expression in PAMs* in vitro* [91], it is proposed that the overrepresented TNF signaling pathway might be the feature of highly pathogenic PRRSV, which needs to be further investigated. In addition to protein-coding genes, a total of 17624 novel and 12987 annotated lncRNAs were obtained from the great amounts of uncharacterized transcripts identified in our study. Zhang et al. characterized 12867 novel lncRNAs during PRRSV infection [19], while our study predicted more novel lncRNAs. Meanwhile we predicted the annotated lncRNAs XR_297549.1 with a highly decreased level during PRRSV infection that was both cis-regulated and trans-regulated by the PTSG2 gene. COX-1 and COX-2 are considered an isoform of cyclooxygenase responsible for the production of prostanoids from arachidonic acid that is hydrolyzed from cell membrane phospholipids by phospholipase A. COX-1 is shown to be expressed constitutively to maintain housekeeping functions, and COX-2 can be induced by multiple stimuli such as bacterial endotoxins lipopolysaccharides (LPS), IL-1, TNF-*α*, and growth factors [92]. Enhanced COX-2 protein levels are associated with the augmented production of its major derivative substrate prostaglandin E_2_ (PGE_2_), leading to the regulation of viral replication and pathological processes of airway inflammation in respiratory diseases [93–95]. Although the interaction between influenza A virus and COX-2 has been extensively studied, the effects of COX-2-induced responses on influenza A virus infection remain controversial [96–100]. HP-PRRSV infection is shown to induce the production of PGE_2_ through COX-1 upregulation, but COX-2 is slightly downregulated at both mRNA and protein levels [101]. A study indicated that both COX-1 and COX-2 mRNA levels were increased upon PRRSV VR-2332 infection [18]. Our data showed that COX-2 gene in PRRSV-infected PAMs was significantly suppressed at all time points. Whether different strains of PRRSV and different multiplicity of infection are related to the gene expression differences and varying degrees of COX-2 should be further explored.

Recent studies have demonstrated that the COX-2 expression can be regulated at different levels, such as transcription, posttranscription, or posttranslation [102]. In addition to RNA-binding proteins, multiple small noncoding RNAs (microRNAs) are involved in COX-2 regulation either directly or indirectly. A newly identified lncRNA, PACER, is shown to promote COX-2 gene expression through occluding its repressor p50 [72]. Although another lncRNA, lncRNA-COX2, has been confirmed to be expressed in similar temporal patterns to its neighboring COX-2 gene in bone marrow-derived dendritic cells (BMDCs) induced by diverse TLRs agonists, it is not involved in COX-2 gene expression [28, 71]. Further studies have illustrated that lncRNA-COX2 mediates both the activation and repression of distinct classes of immune genes via different mechanisms [71, 103, 104]. Comparative analysis showed that the lncRNA XR_297549.1 in* Sus scrofa* was quite different in length, location, and base sequence. Although the predicted XR_297549.1 sequence was removed as a result of standard genome annotation processing in NCBI, our RNA-Seq and further qRT-PCR analysis validated the existence of this transcripts and the significant downregulation phenomenon in response to PRRSV infection. Therefore, further studies are warranted to elucidate the function of lncRNA XR_297549.1 and its correlation with its neighboring COX-2 gene, as well as its role in PRRSV infection.

Our analyses reveal that HP-PRRSV infection triggers dynamic gene expression profiles in PAMs at different time points, indicating the comprehensive interaction between HP-PRRSV and cellular responses. The significant downregulation of essential genes is possibly an essential mechanism for PRRSV to subvert innate and adaptive immune responses of PAMs. Of note, a newly COX-2 neighboring lncRNA XR_297549.1 was discovered to be highly expressed in PAMs and decreased remarkably during PRRSV infection, implicating its potential role in protein-coding genes expression and PRRSV infection. Our findings provide valuable information for further function explorations of mRNAs and lncRNAs with great importance for the pathogenesis of PRRSV.

## Figures and Tables

**Table 1:** Primer used for qRT-PCR validation.

Transcript name	Forward primer (5′-3′)	Reverse primer (5′-3′)	Probe
PRRSV N gene	TCCCTTTAGCGACTGAAGATGAC	TGGATCGACAACAAACACAATTG	CAGGCATCCCTTTACCCCTAGTGAGCG
PPIA	TCTTCTTCGACATCGCCGTC	GCACGGAAGTTTTCTGCTGTCT	CCCTTGGGCCGCGTCTCCTTC
GBP1	ACATGCCCGAACCACAGTG	GATGGCAGACAGGAGCTTCAG	AACATCAATGGGCGACTGGTGGTGA
HPDL	CTGGTTCCACGACTGTCTAGGATTT	GGACTCTGCCAGCACTAGGGTT	ACCTGCCGCTGAGCCCAGGTGA
DPYSL2	CAAGCAAATAGGAGAAAACCTGATT	CTGGAAGCGGGTATGGACA	CAGGAGGGGTGAAGACCATCGAAGC
PRCP	AGGGGACATTATCTGGTTTTGC	GAGGGACTCGCCATAGTATCG	CGGGGTTCATGTGGGATGTAGCTGA
NAAA	ATCCTCCTCAACCTGGCCTAC	AGCATAATCCAGATTCCGGC	CGCATTCTGCACGAGTATTGTGGCT
CD163	CCATTTAAGTTCCTTCACTTTTGCT	TTTCACCACCCGTTAGCCTC	TCGCTGTTCTCAGTGCCTGCTTGGT
XR_301539.1	TTGCATCAAGACCGCTTCTCG	AGCCCCCAAATGTAAGACCAAGA	TAAGGGGAGTCGCCTCTTCCGAGCC
XR_297549.1	AAGACCTGGAGAAAGAGCATTCC	TTTAAATCTCACAGACATGCCTCAG	AGAGGGAAAACAAGTGCAATGGCCC
TCONS_00048171	ATGTCCTTAAATGGCTGCGG	AAAACTGTTCCCAGCCATCTTC	CCCGAACGAGGCAGTCTCCCTTTAT
TCONS_00154605	TTCGGGGTCTGGGACTGAGT	CCATCAGCACCGACACGG	TCCTGCTCCCCTGTCCCCACACCT

**Table 2:** The numbers of differentially expressed mRNAs and lncRNAs of PRRSV-infected PAMs in comparison to mock-infected PAMs.

Comparison group	mRNAs		Annotated lncRNAs		Novel lncRNAs	
	Up	Down	Up	Down	Up	Down
P_V6_ versus P_M_	456	764	118	123	259	180
P_V9_ versus P_M_	2089	1814	632	342	2048	560
P_V12_ versus P_M_	7106	3224	3033	501	6570	992
P_V_ versus P_M_	237	631	67	65	148	93
P_V9_ versus P_V6_	1117	192	84	36	162	113
P_V12_ versus P_V9_	4679	98	190	466	133	283

**Table 3:** Expression dynamics and function prediction of several annotated lncRNAs during PRRSV infection.

Transcript	Gene	P_V6_ versus P_M_			P_V9_ versus P_M_			P_V12_ versus P_M_			Cis-regulated gene	Trans-regulated gene
		Log_2_ (fold change)	Reg	FDR-*p* value	Log_2_ (fold change)	Reg	FDR-*p* value	Log_2_ (fold change)	Reg	FDR-*p* value		
XR_301539.1	LOC102158335	−2.24897	Up	1.27*E* − 86	−2.29946	Up	1.01*E* − 23	−1.37581	Up	1.24*E* − 26	LOC100516661	None
XR_301635.1	LOC102165492	−1.09138	Up	4.17*E* − 15	−1.06908	Up	5.03*E* − 19	−1.04928	Up	1.87*E* − 35	None	None
XR_297549.1	LOC102166377	1.86752	Down	6.75*E* − 55	2.77498	Down	2.12*E* − 11	2.46626	Down	1.72*E* − 78	PTGS2	PTGS2
XR_304346.1	LOC100622791	1.08218	Down	5.51*E* − 23	2.05492	Down	1.60*E* − 11	2.80967	Down	3.93*E* − 13	None	None
XR_299147.1	LOC100624137	1.31202	Down	1.25*E* − 38	2.17241	Down	4.97*E* − 98	2.74384	Down	1.77*E* − 12	None	TMEM254

## Data Availability

The RNA-sequencing data generated in this study have been deposited in the National Center for Biotechnology Information (NCBI) Gene Expression Omnibus (GEO) database with Accession no. GSE89331.

## Supplementary Materials

Supplementary 1 Table S1: the data yields and mapping conditions of each sample in RNA-Seq.

Supplementary 2 Table S2: characteristics of the significantly altered genes involved in viral recognition and cellular responses during PRRSV infection.

Supplementary 3 Table S3: expression dynamics of known antiviral genes during PRRSV infection.

Supplementary 4 Table S4: expression dynamics of potential biomarkers for different macrophage phenotypes during PRRSV infection.

Supplementary 5 Table S5: characteristics of the significantly altered genes involved in antigen processing and presentation during PRRSV infection.

Supplementary 6 Table S6: characteristics of the significantly altered genes involved in phagocytosis during PRRSV infection.
