# Supplementary material for: Transcriptome Analysis Reveals Dynamic Gene Expression Profiles in Porcine Alveolar Macrophages in Response to the Chinese Highly Pathogenic Porcine Reproductive and Respiratory Syndrome Virus
Source: Biomed Res Int. 2018 Apr 29;2018:1538127. doi: 10.1155/2018/1538127 (PMC5949201; doi:10.1155/2018/1538127)
Supplement: Supplementary 1 — Table S1: the data yields and mapping conditions of each sample in RNA-Seq. [file 1538127.f1.doc]

| **Sample name** | **P_M-1_** | **P_M-2_** | **P_V6-1_** | **P_V6-2_** | **P_V9-1_** | **P_V9-2_** | **P_V12-1_** | **P_V12-2_** |
| --- | --- | --- | --- | --- | --- | --- | --- | --- |
| Raw reads | 164,198,830 | 173,044,766 | 131,143,618 | 143,604,792 | 130,256,186 | 129,086,540 | 143,391,752 | 141,879,646 |
| Clean reads* | 147,576,896  (89.88%) | 158,487,162  (91.59%) | 114,617,308  (87.40%) | 124,340,634  (86.59%) | 114,747,254  (88.09%) | 115,519,946  (89.49%) | 128,348,472  (89.51%) | 127,388,996  (89.79%) |
| Total bases | 16,419,883,000 | 17,304,476,600 | 13,114,361,800 | 14,360,479,200 | 13,025,618,600 | 12,908,654,000 | 14,339,175,200 | 14,187,964,600 |
| Clean bases * | 14,522,123,672  (88.44%) | 15,607,726,460  (90.19%) | 11,290,486,235  (86.09%) | 12,267,308,877  (85.42%) | 11,298,495,985  (86.74%) | 11,355,566,393  (87.97%) | 12,666,586,386  (88.34%) | 12,584,324,626  (88.70%) |
| Total mapped* | 125,998,163  (85.66%) | 129,916,358  (83.98) | 97,567,546  (85.28%) | 103,572,304  (83.39%) | 84,198,335  (73.68%) | 83,420,257  (72.32%) | 79,278,137  (61.81%) | 78,497,561  (61.71%) |
|  |  |  |  |  |  |  |  |  |
| Multiple mapped* | 29,157,385  (19.82%) | 28,379,139  (18.34%) | 22,245,255  (19.44%) | 23,448,545  (18.88%) | 20,418,073  (17.87%) | 19,970,536  (17.31%) | 19,766,535  (15.41%) | 19,707,967  (15.49%) |
|  |  |  |  |  |  |  |  |  |
| Uniquely mapped* | 96,840,778  (65.84%) | 101,537,219  (65.63%) | 75,322,291  (65.84%) | 80,123,759  (64.51%) | 63,780,262  (55.81%) | 63,449,721  (55.01%) | 59,511,602  (46.40%) | 58,789,594  (46.21%) |
|  |  |  |  |  |  |  |  |  |
| Reads map to ‘+’ strand* | 62,654,372  (42.60%) | 64,607,779  (41.76%) | 48,433,515  (42.34%) | 51,327,213  (41.32%) | 41,719,705  (36.51%) | 41,398,764  (35.89%) | 39,327,731  (30.66%) | 38,925,898  (30.60%) |
|  |  |  |  |  |  |  |  |  |
| Reads map to ‘-’ strand* | 63,343,79  (43.06%) | 65,308,578  (42.21%) | 49,134,031  (42.95%) | 52,245,091  (42.06%) | 42,478,630  (37.17%) | 42,021,493  (36.43%) | 39,950,406  (31.15%) | 39,571,663  (31.11%) |
|  |  |  |  |  |  |  |  |  |
| Reads mapped in proper pairs | 60,569,824  (41.18%) | 62,107,762  (40.14%) | 46,488,167  (40.63%) | 48,910,721  (39.38%) | 39,898,665  (34.91%) | 39,873,477  (34.57%) | 37,798,769  (29.47%) | 37,383,867  (29.39%) |

Table S1. The data yields and mapping conditions of each sample in RNA-seq
