# Supplementary material for: Transcriptome Analysis Reveals Dynamic Gene Expression Profiles in Porcine Alveolar Macrophages in Response to the Chinese Highly Pathogenic Porcine Reproductive and Respiratory Syndrome Virus
Source: Biomed Res Int. 2018 Apr 29;2018:1538127. doi: 10.1155/2018/1538127 (PMC5949201; doi:10.1155/2018/1538127)
Supplement: Supplementary 2 — Table S2: characteristics of the significantly altered genes involved in viral recognition and cellular responses during PRRSV infection. [file 1538127.f2.doc]

| **Gene** | **Abbr** | **NCBI** | **P_V6_ vs P_M_** | | |  | **P_V9_ vs P_M_** | | |  | **P_V12_ vs P_M_** | | |
| --- | --- | --- | --- | --- | --- | --- | --- | --- | --- | --- | --- | --- | --- |
|  |  |  | **Log_2_ (Fold change)** | **Reg** | **FDR-*p* value** |  | **Log_2_ (Fold change)** | **Reg** | **FDR-*p* value** |  | **Log_2_ (Fold change)** | **Reg** | **FDR-*p* value** |
| Mitogen-activated protein kinase 14 | MAPK14 | XM_001929490.4 | -1.49316 | UP | 8.34E-16 |  | -1.78808 | UP | 5.95E-97 |  | -1.11502 | UP | 5.16E-44 |
| Toll-like receptor 1 | TLR1 | NM_001031775.1 | -1.17492 | UP | 3.65E-25 |  | -1.08945 | UP | 2.73E-12 |  | -0.04532 | UP | 0.498156 |
| Toll-like receptor 6 | TLR6 | NM_213760.1 | -1.22186 | UP | 1.90E-42 |  | -1.07943 | UP | 9.43E-11 |  | 0.059208 | UP | 1.87E-16 |
| Toll-like receptor 8 | TLR8 | NM_214187.1 | 1.623996 | DOWN | 1.37E-30 |  | 1.18336 | DOWN | 5.00E-13 |  | 1.375436 | DOWN | 5.01E-14 |
| Mitogen-activated protein kinase 1 | MAPK1 | NM_001198922.1 | 0.994929 | DOWN | 2.91E-45 |  | 1.487856 | DOWN | 3.61E-102 |  | 2.011951 | DOWN | 2.83E-123 |
| [Phosphoinositide-3-kinase regulatory subunit 5](http://www.ncbi.nlm.nih.gov/gene/23533) | PIK3R5 | NM_213851.1 | 0.620845 | DOWN | 1.90E-17 |  | 1.129013 | DOWN | 1.94E-24 |  | 1.370228 | DOWN | 1.43E-51 |
| [Phosphatidylinositol-4,5-bisphosphate 3-kinase catalytic subunit beta](http://www.ncbi.nlm.nih.gov/gene/5291) | PIK3CB | XM_005669881.1 | 0.744355 | DOWN | 7.38E-22 |  | 1.271259 | DOWN | 3.90E-57 |  | 1.784115 | DOWN | 7.31E-76 |
| [Phosphoinositide-3-kinase regulatory subunit 3](http://www.ncbi.nlm.nih.gov/gene/8503) | PIK3R3 | XM_003128036.4 | 1.222179 | DOWN | 2.06E-05 |  | 1.158293 | DOWN | 1.01E-05 |  | 1.2319 | DOWN | 5.08E-08 |
| [Stathmin 1](http://www.ncbi.nlm.nih.gov/gene/3925) | STMN1 | NM_001009582.1 | 1.674198 | DOWN | 2.50E-59 |  | 2.488688 | DOWN | 2.34E-89 |  | 2.981777 | DOWN | 2.65E-92 |
| Ras-related C3 botulinum toxin substrate 2 (rho family, small GTP binding protein Rac2 | RAC2 | XM_003355342.2 | 1.173105 | DOWN | 4.07E-20 |  | 1.196306 | DOWN | 5.60E-15 |  | 1.561408 | DOWN | 1.32E-22 |
| [Dual specificity phosphatase 4](http://www.ncbi.nlm.nih.gov/gene/1846) | DUSP4 | XM_003133392.2 | 1.919266 | DOWN | 1.61E-59 |  | 2.953729 | DOWN | 4.55E-117 |  | 3.277193 | DOWN | 1.14E-99 |
| Dual specificity phosphatase 6 | DUSP6 | NM_001267842.1 | 0.681532 | DOWN | 6.35E-23 |  | 0.981783 | DOWN | 4.37E-52 |  | 1.924404 | DOWN | 5.23E-126 |

Table S2. Characteristics of the significantly altered genes involved in viral recognition and cellular responses during HP-PRRSV infection
