# Supplementary material for: Transcriptome Analysis Reveals Dynamic Gene Expression Profiles in Porcine Alveolar Macrophages in Response to the Chinese Highly Pathogenic Porcine Reproductive and Respiratory Syndrome Virus
Source: Biomed Res Int. 2018 Apr 29;2018:1538127. doi: 10.1155/2018/1538127 (PMC5949201; doi:10.1155/2018/1538127)
Supplement: Supplementary 3 — Table S3: expression dynamics of known antiviral genes during PRRSV infection. [file 1538127.f3.doc]

Table S3. Expression dynamics of known antiviral genes during PRRSV infection

| **Gene** | **Abbr** | **NCBI** | **P_V6_ vs P_M_** | | |  | **P_V9_ vs P_M_** | | |  | **P_V12_ vs P_M_** | | |
| --- | --- | --- | --- | --- | --- | --- | --- | --- | --- | --- | --- | --- | --- |
|  |  |  | **Log_2_(Fold change)** | **Reg** | **FDR-*p* value** |  | **Log_2_(Fold change)** | **Reg** | **FDR-*p* value** |  | **Log_2_(Fold change)** | **Reg** | **FDR-*p* value** |
| Interferon-induced proteins with tetratricopeptide repeat 1 | IFIT1 | NM_001244363.1 | NS* | NS | NS |  | -3.82492 | UP | 4.45E-59 |  | -6.87031 | UP | 1.44E-15 |
| Interferon-induced proteins with tetratricopeptide repeat 2 | IFIT2 | XM_001928671.3 | NS | NS | NS |  | -3.32613 | UP | 9.86E-96 |  | -4.87492 | UP | 9.27E-19 |
| Interferon-induced proteins with tetratricopeptide repeat 3 | IFIT3 | NM_001204395.1 | NS | NS | NS |  | -2.64287 | UP | 1.69E-29 |  | -4.46313 | UP | 1.26E-11 |
| Interferon-induced proteins with tetratricopeptide repeat 5 | IFIT5 | XM_001925952.3 | NS | NS | NS |  | -1.82888 | UP | 3.70E-85 |  | -3.50456 | UP | 1.53E-65 |
| Interferon-induced transmembrane protein 1 | IFITM1 | XM_003124230.2 | 1.276652 | DOWN | 1.08E-30 |  | NS | NS | NS |  | NS | NS | NS |
| Interferon-induced transmembrane protein 2 | IFITM2 | NM_001246214.1 | NS | NS | NS |  | NS | NS | NS |  | -1.00701 | DOWN | 9.81E-07 |
| Interferon-induced transmembrane protein 3 | IFITM3 | NM_001201382.1 | NS | NS | NS |  | NS | NS | NS |  | -1.61477 | DOWN | 1.28E-09 |
| Tripartite motif protein 14 | TRIM14 | XM_003122031.2 | NS | NS | NS |  | NS | NS | NS |  | -1.14818 | UP | 9.39E-17 |
| Tripartite motif protein 21 | TRIM21 | NM_001163649.2 | NS | NS | NS |  | -1.02421 | UP | 4.87E-20 |  | -2.48135 | UP | 2.77E-56 |
| Myxovirus resistance 1 | MX1 | NM_214061.1 | NS | NS | NS |  | -1.14952 | UP | 3.86E-10 |  | -2.26495 | UP | 2.58E-19 |
| Interferon stimulated gene 15 | ISG15 | NM_001128469.1 | NS | NS | NS |  | NS | NS | NS |  | -1.06851 | UP | 1.30E-44 |
| Interferon stimulated gene 20 | ISG20 | XM_005660030.1 | NS | NS | NS |  | -1.95721 | UP | 1.16E-13 |  | -4.73801 | UP | 1.23E-21 |
| Cholesterol-25-hydroxylase | CH25H | XM_001928620.3 | NS | NS | NS |  | -1.69107 | UP | 5.11E-19 |  | -2.61216 | UP | 8.59E-42 |
| Double-stranded RNA-activated protein kinase | PKR | NM_214319.1 | NS | NS | NS |  | NS | NS | NS |  | -1.75598 | UP | 2.25E-78 |
| Tetherin | BST2 | NM_001161755.1 | NS | NS | NS |  | NS | NS | NS |  | -2.26334 | UP | 1.74E-33 |
| Guanylate binding protein 1 | GBP1 | NM_001128473.1 | -2.019603 | UP | 1.34E-12 |  | -3.09643 | UP | 2.22E-12 |  | -4.40673 | UP | 3.54E-53 |

*NS means no significant
