# Supplementary material for: Transcriptome Analysis Reveals Dynamic Gene Expression Profiles in Porcine Alveolar Macrophages in Response to the Chinese Highly Pathogenic Porcine Reproductive and Respiratory Syndrome Virus
Source: Biomed Res Int. 2018 Apr 29;2018:1538127. doi: 10.1155/2018/1538127 (PMC5949201; doi:10.1155/2018/1538127)
Supplement: Supplementary 4 — Table S4: expression dynamics of potential biomarkers for different macrophage phenotypes during PRRSV infection. [file 1538127.f4.doc]

| TableS4. Expression dynamics of potential biomarkers for different macrophage phenotypes during HP-PRRSV infection | | | | | | | | | | | | | |
| --- | --- | --- | --- | --- | --- | --- | --- | --- | --- | --- | --- | --- | --- |
| **Gene** | **Abbr** | **NCBI** | **P_V6_ vs P_M_** | | |  | **P_V9_ vs P_M_** | | |  | **P_V12_ vs P_M_** | | |
|  |  |  | **Log_2_(Fold change)** | **Reg** | **FDR-*p* value** |  | **Log_2_(Fold change)** | **Reg** | **FDR-*p* value** |  | **Log_2_(Fold change)** | **Reg** | **FDR-*p* value** |
| M1 phenotype |  |  |  |  |  |  |  |  |  |  |  |  |  |
| Receptor |  |  |  |  |  |  |  |  |  |  |  |  |  |
| Swine leukocyte antigen class II DM alpha domain | SLA-DMA | NM_001004039.1 | NS | NS | NS |  | 1.45293 | DOWN | 5.13E-1 |  | 1.55724 | DOWN | 3.89E-26 |
| Swine leukocyte antigen class II DM beta domain | SLA-DMB | NM_001113707.1 | NS | NS | NS |  | NS | NS | NS |  | 1.03469 | DOWN | 7.14E-14 |
| Swine leukocyte antigen class II DO alpha domain | SLA-DOA | NM_001185143.1 | 1.01827 | DOWN | 2.52E-22 |  | 1.18301 | DOWN | 2.08E-26 |  | 1.69624 | DOWN | 7.26E-41 |
| Swine leukocyte antigen class II DO beta domain | SLA-DOB | NM_001114064.2 | -1.00546 | UP | 0.41508 |  | -2.57567 | UP | 0.00139 |  | -3.65261 | UP | 1.99E-08 |
| Swine leukocyte antigen class II DQ alpha1 domain | SLA-DQA1 | NM_001114062.2 | 1.26438 | DOWN | 7.39E-47 |  | 2.02893 | DOWN | 4.35E-19 |  | 2.52443 | DOWN | 2.74E-18 |
| Swine leukocyte antigen class II DQ beta1 domain | SLA-DQB1 | NM_001113694.1 | 1.45364 | DOWN | 5.42E-65 |  | 2.20118 | DOWN | 3.18E-18 |  | 2.62198 | DOWN | 1.79E-17 |
| Swine leukocyte antigen class II DR alpha domain | SLA-DRA | NM_001113706.1 | NS | NS | NS |  | 1.40022 | DOWN | 4.20E-84 |  | 1.61064 | DOWN | 2.54E-73 |
| Swine leukocyte antigen class II DR beta1 domain | SLA-DRB1 | NM_001113694.1 | 1.72053 | DOWN | 2.39E-49 |  | 2.56774 | DOWN | 9.56E-23 |  | 3.23147 | DOWN | 2.30E-23 |
| Cytokine |  |  |  |  |  |  |  |  |  |  |  |  |  |
| Interleukin 6 | IL-6 | NM_214399.1 | 2.13253 | DOWN | 0.0066 |  | 2.39484 | DOWN | 1.81E-06 |  | 2.72647 | DOWN | 0.00187 |
| Interleukin 12A | IL-12A | NM_213993.1 | NS | NS | NS |  | NS | NS | NS |  | -1.75901 | UP | 0.00133 |
| Interleukin 12B | IL-12B | NM_214013.1 | NS | NS | NS |  | NS | NS | NS |  | NS | NS | NS |
| Interleukin 15 | IL-15 | NM_214390.1 | NS | NS | NS |  | -1.40369 | UP | 0.00053 |  | -3.41577 | UP | 1.69E-34 |
| Interleukin 1β | IL-1β | NM_001005149.1 | NS | NS | NS |  | NS | NS | NS |  | NS | NS | NS |
| Tumor necrosis factor α | TNF-α | NM_214022.1 | 1.31146 | DOWN | 8.84E-87 |  | 1.16781 | DOWN | 1.14E-66 |  | NS | NS | NS |
| Chemokine |  |  |  |  |  |  |  |  |  |  |  |  |  |
| C-C motif chemokine 8 | CCL8 | NM_001164515.1 | NS | NS | NS |  | -3.38183 | UP | 8.25E-73 |  | -5.59767 | UP | 3.20E-29 |
| C-C motif chemokine 20 | CCL20 | NM_001024589.1 | 1.09814 | DOWN | 3.90E-07 |  | 1.10075 | DOWN | 8.91E-05 |  | -1.48099 | UP | 1.64E-19 |
| Chemokine (C-X-C motif) ligand 10 | CXCL10 | NM_001008691.1 | 1.29421 | DOWN | 0.00063 |  | -5.35939 | UP | 2.39E-11 |  | -8.38734 | UP | 4.11E-77 |
| Metabolic factor |  |  |  |  |  |  |  |  |  |  |  |  |  |
| Nitric oxide synthase 1 adaptor protein | NOS1AP | XM_005674585.1 | -2.12155 | UP | 0.00988 |  | -1.86667 | UP | 0.03514 |  | -3.46758 | UP | 6.58E-09 |
| Nitric oxide synthase 3 | NOS3 | NM_214295.1 | NS | NS | NS |  | NS | NS | NS |  | -3.10232 | UP | 1.86E-11 |
| Prostaglandin-endoperoxide synthase 2 | PTGS2 | NM_214321.1 | 1.92886 | DOWN | 6.39E-90 |  | 2.93615 | DOWN | 1.60E-15 |  | 2.23138 | DOWN | 1.50E-86 |
|  |  |  |  |  |  |  |  |  |  |  |  |  |  |
| M2a phenotype |  |  |  |  |  |  |  |  |  |  |  |  |  |
| Receptor |  |  |  |  |  |  |  |  |  |  |  |  |  |
| Class A scanvenger receptor | SR-A1 | XM_003124013.2 | NS | NS | NS |  | NS | NS | NS |  | 1.24158 | DOWN | 1.17E-25 |
| Cluster of Differentiation 302 | CD302 | NM_001110425.1 | NS | NS | NS |  | NS | NS | NS |  | 1.99989 | DOWN | 0.00294 |
| C-type lectin domain family 5 member A | CLEC5A | NM_213990.1 | NS | NS | NS |  | 1.70002 | DOWN | 0.00199 |  | 1.55725 | DOWN | 0.00483 |
| Cytokine |  |  |  |  |  |  |  |  |  |  |  |  |  |
| Transforming growth factor beta 1 | TGF-β1 | NM_214015.1 | NS | NS | NS |  | 1.13182 | DOWN | 6.70E-48 |  | 1.34027 | DOWN | 1.15E-49 |
| Transforming growth factor beta 2 | TGF-β2 | XM_005653762.1 | NS | NS | NS |  | NS | NS | NS |  | -1.97726 | UP | 9.75E-10 |
| Chemokine |  |  |  |  |  |  |  |  |  |  |  |  |  |
| C-C motif chemokine 14 | CCL14 | XM_005656979.1 | 1.46975 | DOWN | 0.13398 |  | 1.48861 | DOWN | 0.09911 |  | NS | NS | NS |
| C-C motif chemokine 23 | CCL23 | XM_003131712.4 | 1.67743 | DOWN | 4.17E-72 |  | 2.63313 | DOWN | 1.88E-26 |  | 3.08087 | DOWN | 6.07E-27 |
| Metabolic factor |  |  |  |  |  |  |  |  |  |  |  |  |  |
| Arginase | Arg1 | NM_214048.2 | 1.32681 | DOWN | 1.30E-21 |  | 2.47721 | DOWN | 2.97E-97 |  | 2.99694 | DOWN | 9.46E-89 |
|  |  |  |  |  |  |  |  |  |  |  |  |  |  |
| M2b phenotype |  |  |  |  |  |  |  |  |  |  |  |  |  |
| Cytokine |  |  |  |  |  |  |  |  |  |  |  |  |  |
| Interleukin 10 | IL-10 | NM_214041.1 | NS | NS | NS |  | NS | NS | NS |  | -1.31482 | UP | 2.21E-07 |
| Chemokine |  |  |  |  |  |  |  |  |  |  |  |  |  |
| Chemokine (C-X-C motif) ligand 2 | CXCL2 | NM_001001861.2 | NS | NS | NS |  | 1.34515 | DOWN | 6.28E-53 |  | 1.31127 | DOWN | 3.41E-32 |
| Metabolic factor |  |  |  |  |  |  |  |  |  |  |  |  |  |
| Sphingosine kinase 1 | SPHK1 | XM_005656915.1 | 1.14522 | DOWN | 0.00024 |  | 1.34042 | DOWN | 1.11E-08 |  | NS | NS | NS |
|  |  |  |  |  |  |  |  |  |  |  |  |  |  |
| M2c phenotype |  |  |  |  |  |  |  |  |  |  |  |  |  |
| Receptor |  |  |  |  |  |  |  |  |  |  |  |  |  |
| Cluster of Differentiation 163 | CD163 | NM_213976.1 | 2.48246 | DOWN | 1.93E-22 |  | 3.15504 | DOWN | 2.39E-27 |  | 3.72725 | DOWN | 4.24E-30 |
| Toll-like receptor 8 | TLR8 | NM_214187.1 | 1.66474 | DOWN | 3.62E-26 |  | 1.16962 | DOWN | 5.00E-13 |  | 1.31038 | DOWN | 5.01E-14 |
| Toll-like receptor 1 | TLR1 | NM_001031775.1 | -1.17666 | UP | 3.65E-25 |  | -1.10287 | UP | 2.73E-12 |  | NS | NS | NS |
| Interleukin 21 receptor | IL21R | XM_003124527.2 | NS | NS | NS |  | NS | NS | NS |  | -1.28489 | UP | 0.00065 |

*NS means no significant
