# Supplementary material for: Transcriptome Analysis Reveals Dynamic Gene Expression Profiles in Porcine Alveolar Macrophages in Response to the Chinese Highly Pathogenic Porcine Reproductive and Respiratory Syndrome Virus
Source: Biomed Res Int. 2018 Apr 29;2018:1538127. doi: 10.1155/2018/1538127 (PMC5949201; doi:10.1155/2018/1538127)
Supplement: Supplementary 5 — Table S5: characteristics of the significantly altered genes involved in antigen processing and presentation during PRRSV infection. [file 1538127.f5.doc]

Table S5. Characteristics of the significantly altered genes involved in antigen processing and presentation during PRRSV infection

| **Gene** | **Abbr** | **NCBI** | **P_V6_ vs P_M_** | | |  | **P_V9_ vs P_M_** | | |  | **P_V12_ vs P_M_** | | |
| --- | --- | --- | --- | --- | --- | --- | --- | --- | --- | --- | --- | --- | --- |
|  |  |  | **Log2(Fold change)** | **Reg** | **FDR-*p* value** |  | **Log2(Fold change)** | **Reg** | **FDR-*p* value** |  | **Log2(Fold change)** | **Reg** | **FDR-*p* value** |
| Swine leukocyte antigen class 1 | SLA-1 | XM_005665686.1 | 0.805663 | DOWN | 4.03E-23 |  | 1.587303 | DOWN | 8.34E-113 |  | 1.840725 | DOWN | 4.45E-95 |
| Swine leukocyte antigen class 3 | SLA-3 | NM_001097427.1 | 0.928237 | DOWN | 3.50E-34 |  | 1.54011 | DOWN | 9.08E-122 |  | 1.724637 | DOWN | 6.34E-98 |
| Swine leukocyte antigen class 5 | SLA-5 | XM_005665688.1 | 0.580881 | DOWN | 0.394067 |  | 1.782919 | DOWN | 1.17E-33 |  | 2.044585 | DOWN | 9.91E-43 |
| Swine leukocyte antigen class 6 | SLA-6 | NM_001113704.1 | 0.791378 | DOWN | 3.32E-11 |  | 1.285027 | DOWN | 2.01E-24 |  | 1.541759 | DOWN | 7.06E-26 |
| Swine leukocyte antigen class 7 | SLA-7 | NM_213768.1 | 0.553631 | DOWN | 1.69E-12 |  | 1.210405 | DOWN | 4.64E-57 |  | 1.09251 | DOWN | 7.11E-33 |
| Swine leukocyte antigen class 8 | SLA-8 | NM_001113703.1 | 0.679597 | DOWN | 4.87E-10 |  | 0.983626 | DOWN | 8.92E-52 |  | 1.253155 | DOWN | 2.14E-21 |
| [beta-2-microglobulin](http://www.ncbi.nlm.nih.gov/gene/567) | B2m | NM_213978.1 | 0.713002 | DOWN | 0.6177 |  | 7.422076 | DOWN | 0.08550 |  | 7.974955 | DOWN | 1.91E-06 |
| Swine leukocyte antigen class II DM alpha domain | SLA-DMA | NM_001004039.1 | 0.747339 | DOWN | 2.25E-09 |  | 1.466346 | DOWN | 5.13E-19 |  | 1.622102 | DOWN | 3.89E-26 |
| Swine leukocyte antigen class II DM beta domain | SLA-DMB | NM_001113707.1 | 0.774199 | DOWN | 6.28E-28 |  | 0.843695 | DOWN | 2.83E-26 |  | 1.099572 | DOWN | 7.14E-14 |
| Swine leukocyte antigen class II DO alpha domain | SLA-DOA | NM_001185143.1 | 1.020092 | DOWN | 2.52E-22 |  | 1.196612 | DOWN | 2.08E-26 |  | 1.761196 | DOWN | 7.26E-41 |
| Swine leukocyte antigen class II DQ alpha1 domain | SLA-DQA1 | NM_001114062.2 | 1.26615 | DOWN | 1.84E-41 |  | 2.04245 | DOWN | 4.35E-187 |  | 2.589341 | DOWN | 2.74E-182 |
| Swine leukocyte antigen class II DQ beta1 domain | SLA-DQB1 | NM_001113694.1 | 1.45541 | DOWN | 5.42E-65 |  | 2.214697 | DOWN | 3.18E-183 |  | 2.686883 | DOWN | 1.79E-172 |
| Swine leukocyte antigen class II DR alpha domain | SLA-DRA | NM_001113706.1 | 0.898941 | DOWN | 1.71E-28 |  | 1.413745 | DOWN | 4.20E-84 |  | 1.67556 | DOWN | 2.54E-73 |
| Swine leukocyte antigen class II DR beta1 domain | SLA-DRB1 | NM_001113695.1 | 1.688021 | DOWN | 1.23E-54 |  | 2.573746 | DOWN | 9.56E-226 |  | 3.296377 | DOWN | 1.04E-219 |
| Protein disulfide-isomerase A3 | PDIA3 | NM_001195112.1 | 1.179282 | DOWN | 1.01E-68 |  | 1.689473 | DOWN | 2.35E-131 |  | 1.896912 | DOWN | 6.72E-113 |
| Gamma-interferon-inducible lysosomal thiol reductase | IFI30 | NM_001131046.1 | 1.087401 | DOWN | 1.08E-30 |  | 1.707387 | DOWN | 2.07E-151 |  | 2.01475 | DOWN | 2.01E-136 |
