# Supplementary material for: Transcriptome Analysis Reveals Dynamic Gene Expression Profiles in Porcine Alveolar Macrophages in Response to the Chinese Highly Pathogenic Porcine Reproductive and Respiratory Syndrome Virus
Source: Biomed Res Int. 2018 Apr 29;2018:1538127. doi: 10.1155/2018/1538127 (PMC5949201; doi:10.1155/2018/1538127)
Supplement: Supplementary 6 — Table S6: characteristics of the significantly altered genes involved in phagocytosis during PRRSV infection. [file 1538127.f6.doc]

Table S6. Characteristics of the significantly altered genes involved in phagocytosis during PRRSV infection

| **Gene** | **Abbr** | **NCBI** | **P_V6_ vs P_M_** | | |  | **P_V9_ vs P_M_** | | |  | **P_V12_ vs P_M_** | | |
| --- | --- | --- | --- | --- | --- | --- | --- | --- | --- | --- | --- | --- | --- |
|  |  |  | **Log2 (Fold change)** | **Reg** | **FDR-*p* value** |  | **Log2 (Fold change)** | **Reg** | **FDR-*p* value** |  | **Log2 (Fold change)** | **Reg** | **FDR-*p* value** |
| Beta-actin | ACTB | XM_003124280.3 | 1.219072 | DOWN | 3.38E-50 |  | 1.619772 | DOWN | 1.17E-100 |  | 1.850363 | DOWN | 6.65E-81 |
| Ras-related protein Rab-5A | RAB5A | NM_001123180.1 | 0.68459 | DOWN | 1.84E-05 |  | 1.036949 | DOWN | 8.78E-30 |  | 1.245603 | DOWN | 8.64E-31 |
| Ras-related protein Rab-5C | RAB5C | NM_001244254.1 | 0.773211 | DOWN | 5.88E-23 |  | 0.973844 | DOWN | 1.39E-47 |  | 1.124026 | DOWN | 9.73E-42 |
| Calreticulin | CALR | NM_001174133.1 | 0.75547 | DOWN | 1.09E-18 |  | 1.207666 | DOWN | 7.35E-72 |  | 1.507135 | DOWN | 1.69E-70 |
| Lysosomal-associated membrane protein 1 | LAMP1 | XM_005668552.1 | 1.199675 | DOWN | 4.44E-73 |  | 1.34387 | DOWN | 1.12E-53 |  | 1.440899 | DOWN | 4.89E-71 |
| mannose 6-phosphate receptor | M6PR | XM_001927611.3 | 0.875789 | DOWN | 7.63E-26 |  | 1.429415 | DOWN | 4.72E-103 |  | 1.756826 | DOWN | 3.34E-99 |
| High affinity immunoglobulin gamma Fc receptor I | FCGR1A | XM_005663509.1 | 2.178906 | DOWN | 2.92E-159 |  | 2.617336 | DOWN | 0.459358 |  | 2.445959 | DOWN | 0.018894 |
| Integrin alpha-V | ITGAV | NM_001083932.1 | 0.814747 | DOWN | 2.45E-15 |  | 1.283953 | DOWN | 6.61E-38 |  | 1.698097 | DOWN | 1.43E-47 |
| Integrin alpha-5 | ITGA5 | XM_001925252.5 | 1.062098 | DOWN | 9.68E-52 |  | 1.852253 | DOWN | 1.19E-136 |  | 1.961875 | DOWN | 7.37EE-108 |
| Integrin beta-1 | ITGB1 | XM_005668173.1 | 0.69327 | DOWN | 1.26E-16 |  | 1.100972 | DOWN | 1.22E-66 |  | 1.542946 | DOWN | 1.13E-84 |
| Integrin beta-2 | ITGB2 | NM_213908.1 | 0.916398 | DOWN | 7.50E-28 |  | 1.230469 | DOWN | 3.35E-73 |  | 1.540778 | DOWN | 2.22E-74 |
| Complement component 3 | C3 | NM_214009.1 | 1.200774 | DOWN | 1.15E-64 |  | 2.571448 | DOWN | 4.43E-270 |  | 3.418625 | DOWN | 7.97E-16 |
| Toll-like receptor 2 | TLR2 | XM_005653576.1 | 0.332513 | DOWN | 0.319757 |  | 0.806821 | DOWN | 2.39E-35 |  | 1.33952 | DOWN | 1.37E-63 |
| C-type lectin domain family 7 member A | CLEC7A | NM_001145866.1 | 1.207294 | DOWN | 4.30E-70 |  | 1.789909 | DOWN | 7.96E-46 |  | 2.058774 | DOWN | 2.95E-116 |
| Macrophage scavenger receptor 1 | MSR1 | NM_001243874.1 | 1.229767 | DOWN | 1.13E-36 |  | 2.337684 | DOWN | 1.92E-204 |  | 3.008968 | DOWN | 3.99E-224 |
| Oxidized low-density lipoprotein receptor 1 | OLR1 | NM_213805.1 | 1.738701 | DOWN | 5.87E-35 |  | 2.526853 | DOWN | 1.34E-48 |  | 2.336018 | DOWN | 4.87E-41 |
| [cluster of differentiation](https://en.wikipedia.org/wiki/Cluster_of_differentiation) 36 | CD36 | XM_005667691.1 | 0.786453 | DOWN | 3.11E-24 |  | 1.356948 | DOWN | 7.87E-95 |  | 1.874676 | DOWN | 1.67E-113 |
| Cathepsin A | CTSA | NM_001243629.1 | 1.052578 | DOWN | 2.16E-58 |  | 1.735067 | DOWN | 5.21E-160 |  | 2.240502 | DOWN | 1.79E-173 |
| Cathepsin B | CTSB | XM_005657265.1 | 0.540536 | DOWN | 0.000367 |  | 1.325641 | DOWN | 9.09E-36 |  | 1.847843 | DOWN | 6.81E-43 |
| Cathepsin D | CTSD | NM_001037721.1 | 1.745688 | DOWN | 2.44E-139 |  | 2.630361 | DOWN | 6.06E-32 |  | 3.09098 | DOWN | 1.60E-288 |
| Cathepsin H | CTSH | NM_213929.2 | 0.439701 | DOWN | 1.29E-06 |  | 0.653184 | DOWN | 6.94E-21 |  | 0.773234 | DOWN | 2.19E-17 |
| Cathepsin Z | CTSZ | NM_001123104.1 | 1.553466 | DOWN | 7.35E-102 |  | 1.6832 | DOWN | 9.34E-114 |  | 2.034883 | DOWN | 1.16E-113 |
| Lysosomal alpha-glucosidase | GAA | XM_003482942.2 | 1.088505 | DOWN | 1.38E-56 |  | 1.468295 | DOWN | 3.26E-96 |  | 1.682776 | DOWN | 5.77E-86 |
| Hexosaminidase A | HEXA | NM_001123221.1 | 1.40806 | DOWN | 8.54E-82 |  | 1.943608 | DOWN | 1.46E-194 |  | 2.088804 | DOWN | 5.08E-151 |
| Hexosaminidase B | HEXB | NM_213921.1 | 1.159189 | DOWN | 4.14E-58 |  | 1.91396 | DOWN | 1.05E-172 |  | 2.321491 | DOWN | 8.29E-170 |
| Beta-mannosidase 1 | MAN2B1 | XM_003123314.2 | 1.884341 | DOWN | 4.07E-174 |  | 2.897439 | DOWN | 8.67E-16 |  | 3.445515 | DOWN | 3.19E-33 |
| Arylsulfatase B | ARSB | XM_005661511.1 | 0.635344 | DOWN | 8.01E-06 |  | 1.33883 | DOWN | 1.32E-30 |  | 1.792235 | DOWN | 1.50E-34 |
| Arylsulfatase G | ARSG | XM_003131263.2ARSG | 0.728182 | DOWN | 2.44E-10 |  | 1.428401 | DOWN | 3.59E-26 |  | 1.224186 | DOWN | 4.57E-17 |
| Lysosomal lipase | LIPA | XM_005671253.1 | 1.670627 | DOWN | 5.56E-129 |  | 2.173336 | DOWN | 2.05E-200 |  | 2.446991 | DOWN | 2.07E-84 |
| Deoxyribonuclease II | Dnase2 | NM_214196.1 | 1.263215 | DOWN | 1.94E-79 |  | 1.920129 | DOWN | 2.28E-167 |  | 2.27006 | DOWN | 1.63E-158 |
| N(4)-(beta-N-acetylglucosaminyl)-L-asparaginas | AGA | XM_001927332.5 | 1.257947 | DOWN | 9.49E-37 |  | 1.778463 | DOWN | 1.10E-58 |  | 2.194907 | DOWN | 3.49E-66 |
| GM2 ganglioside activator | GM2A | XM_003134142.4 | 1.191551 | DOWN | 1.53E-55 |  | 1.592927 | DOWN | 1.10E-114 |  | 1.665241 | DOWN | 1.46E-86 |
| Palmitoyl-protein thioesterase 1 | PPT1 | XM_003356329.2 | 1.865026 | DOWN | 4.24E-113 |  | 2.354061 | DOWN | 8.98E-155 |  | 2.805751 | DOWN | 7.44E-155 |
| Lysosomal-associated transmembrane protein 4A | LAPTM4A | XM_005662724.1 | 0.575594 | DOWN | 2.77E-13 |  | 1.010666 | DOWN | 6.04E-56 |  | 0.806601 | DOWN | 8.79E-23 |
| Lysosomal-associated transmembrane protein 4B | LAPTM4B | XM_005655356.1 | 1.026646 | DOWN | 5.99E-12 |  | 1.590789 | DOWN | 3.99E-22 |  | 1.903252 | DOWN | 7.22E-25 |
| Lysosomal-associated transmembrane protein 5 | LAPTM5 | XM_003356262.3 | 1.015671 | DOWN | 9.17E-48 |  | 1.625885 | DOWN | 1.17E-130 |  | 2.08837 | DOWN | 1.79E-137 |
| ATP-binding cassette sub-family B member 9 | ABCB9 | XM_005670603.1 | 1.456297 | DOWN | 1.01E-17 |  | 1.561728 | DOWN | 3.27E-18 |  | 1.667749 | DOWN | 2.87E-17 |
| Sortilin 1 | SORT1 | XM_005663614.1 | 1.483104 | DOWN | 9.87E-52 |  | 2.326622 | DOWN | 2.50E-96 |  | 2.519091 | DOWN | 1.76E-87 |
| V-type proton ATPase 16 kDa proteolipid subunit | ATP6V0C | XM_003124744.2 | 0.655333 | DOWN | 1.23E-08 |  | 1.073018 | DOWN | 1.53E-14 |  | 1.231519 | DOWN | 1.71E-15 |
| V-type proton ATPase subunit d 2 | ATP6V0D2 | XM_003125581.2 | 0.990131 | DOWN | 1.60E-08 |  | 1.714117 | DOWN | 2.33E-21 |  | 2.137464 | DOWN | 1.60E-25 |
| V-type proton ATPase subunit d 1 | ATP6V0D1 | XM_003126946.2 | 0.937789 | DOWN | 9.47E-33 |  | 1.394784 | DOWN | 3.90E-105 |  | 1.605755 | DOWN | 1.20E-90 |
| V-type proton ATPase subunit S1 | ATP6AP1 | XM_005674059.1 | 2.194453 | DOWN | 1.28E-36 |  | -0.79756 | DOWN | 2.18E-62 |  | 3.878689 | DOWN | 2.76E-89 |
| V-type proton ATPase subunit H | ATP6V1H | NM_214240.1 | 0.414557 | DOWN | 3.03E-07 |  | 0.571055 | DOWN | 2.61E-17 |  | 0.59978 | DOWN | 7.11E-12 |
| V-type proton ATPase 116 kDa subunit a isoform 3 | TCIRG1 | XM_005660613.1 | 0.720626 | DOWN | 1.11E-19 |  | 0.865818 | DOWN | 7.96E-42 |  | 0.859515 | DOWN | 9.57E-26 |
